# HistoneH3 demethylase JMJD2A promotes growth of liver cancer cells through up-regulating miR372

**DOI:** 10.18632/oncotarget.17095

**Published:** 2017-04-13

**Authors:** Jiahui An, Jie Xu, Jiao Li, Song Jia, Xiaonan Li, Yanan Lu, Yuxin Yang, Zhuojia Lin, Xiaoru Xin, Mengying Wu, Qidi Zheng, Hu Pu, Xin Gui, Tianming Li, Dongdong Lu

**Affiliations:** ^1^ School of Life Science and Technology, Tongji University, Shanghai, 20092, China; ^2^ School of Medicine, Tongji University, Shanghai, 200092, China

**Keywords:** liver cancer, HistoneH3 demethylase JMJD2A, miR372, P21WAF1/Cip1, Pim1

## Abstract

Changes in histone lysine methylation status have been observed during cancer formation. JMJD2A protein is a demethylase that is overexpressed in several tumors. Herein, our results demonstrate that JMJD2A accelerates malignant progression of liver cancer cells *in vitro* and *in vivo*. Mechanistically, JMJD2A promoted the expression and mature of pre-miR372 epigenetically. Notably, miR372 blocks the editing of 13th exon-introns-14th exon and forms a novel transcript(JMJD2AΔ) of JMJD2A. In particular, JMJD2A inhibited P21(WAF1/Cip1) expression by decreasing H3K9me3 dependent on JMJD2AΔ. Thereby, JMJD2A could enhance Pim1 transcription by suppressing P21(WAF1/Cip1). Furthermore, through increasing the expression of Pim1, JMJD2A could facilitate the interaction among pRB, CDK2 and CyclinE which prompts the transcription and translation of oncogenic C-myc. Strikingly, JMJD2A may trigger the demethylation of Pim1. On the other hand, Pim1 knockdown and P21(WAF1/Cip1) overexpression fully abrogated the oncogenic function of JMJD2A. Our observations suggest that JMJD2A promotes liver cancer cell cycle progress through JMJD2A-miR372-JMJD2AΔ-P21WAF1/Cip1-Pim1-pRB-CDK2-CyclinE-C-myc axis. This study elucidates a novel mechanism for JMJD2A in liver cancer cells and suggests that JMJD2A can be used as a novel therapeutic targets of liver cancer.

## INTRODUCTION

It is well known that Histone modifications control chromatin function. JMJD2A belongs to a demethylase for histone H3 on lysine 9/36. JMJD2A is overexpressed in cancer [1] and inhibits repair of DNA damage by reducing homologous recombination (HR) repair [2, 3]. Strikingly, JMJD2A is negatively regulated by SIRT2 in cancer [4] and is enhanced via Hypoxia to provide unmethylated H3K9 residues [5]. Of significance, JMJD2A level is negatively associated with miR-34a and JMJD2A inhibits the output of miR-34a [6].

Recently, microRNA-372 (miR372) has been proved to play a substantial role in several human cancers [7]. For examples, upregulation of miR372 promotes metastasis of oral and liver cancer [8, 9]. Importantly, miR372 activated the NFκB signaling and autophagy in cancer cells [10, 11]. Moreover, a miR372/let-7 axis regulates human somatic cell and cancer cell fates [12, 13]. Studies indicates that miR372 inhibits the Ras homolog gene family member C (RhoC) [14] and p62 in human cancer cells [15]. However, the tumor suppressing roles of miR372 was also found in some cancer cells, possibly via the down-regulation of CDK2 and CCNA1 [16].

PIM kinases (a family of Ser/Thr kinases) promotes proliferation and progression of cancer cell [17]. Our previous study showed that upregulation of Pim1 promotes hepatocarcinogenesis through PKM2 and CUDR [18]. Moreover, the Pim1 kinase promotes migration and adhesion of cancer cell [19]. Importantly, Pim1 promotes chemoresistance in cancer [20].

Moreover, HistoneH3K36 trimethylation (H3K36me3) is associated with carcinogenesis [21, 22]. Argonaute proteins AGOI is involved in microRNA -mediated processes, specifically, in cancer cells [23]. Furthermore, heterochromatin protein 1 (HP1) plays versatile functions dyring chromatin remodeling, DNA repair, senescence [24]. Specifically, H2A.Z cooperates with heterochromatin factors to regulate gene expression [25]. In additional, abnornal expression of the DNA methyltransferases (DNMTs) are closely associated with carcinogenesis [26, 27]. Specifically, DNMT3b regulates expression of mTORC2 in cancer cells [28]. On the other hand, oncogenic EGFR inhibits the activity of demethylase in cancer [29, 30].

In this study, we indicate that JMJD2A accelerates malignant progression of liver cancer cells. Moreover, JMJD2A inhibited P21(WAF1/Cip1) and enhanced Pim1 through JMJD2AΔ dependent on miR372. This study elucidates a novel mechanism for JMJD2A in liver cancer cells and suggests that JMJD2A can be used as a novel therapeutic targets for liver cancer.

### Experimental material and procedures

#### Cell lines and plasmids

Human hepatoma cell lines Hep3B was obtained from the Cell Bank of Chinese Academy of Sciences (Shanghai, China). These cell lines were maintained in Dulbecco's modified Eagle medium (Gibco BRL Life Technologies) supplemented with 10% fetal bovine serum (Gibco BRL Life Technologies) in a humidified atmosphere of 5% CO_2_ incubator at 37°C. pCMV6-AC-GFP, pCMV6-AC-GFP-JMJD2A, pGFP-V-RS, pGFP-V-RS-Pim1 were purchased from Origene (Rockville, MD, USA) and pcDNA3.1, pcDNA3.1-P21WAF1/Cip1 were purchased from Addgene (Cambridge, MA, USA).

#### RT-PCR

Total RNA was purified using Trizol (Invitrogen) according to manufacturer's instructions. cDNA was prepared by using oligonucleotide (dT)_15_, random primers, and a SuperScript First-Strand Synthesis System (Invitrogen). PCR analysis was performed under the specical conditions. β-actin was used as an internal control.

#### MicroRNA detection

Total RNA was isolated from cultured cells using Trizol (Invitrogen, Carlsbad, CA, USA) according to the manufacturer's protocol. Real-time RT-PCR-based detection of mature miR372 and U6 snRNA was achieved with the miRNA Detection kit (including a universe primer, U6 primers, Qiagen) and miR372 specific upsteam primers (Origene, USA). qRT-PCR was performed with a StepOnePlus real-time PCR system (Applied Biosystems), a SuperScript First-Strand Synthesis System (Invitrogen, Carlsbad, CA, USA) and Power SYBR Green PCR Master Mix (Applied Biosystems) in accordance with the manufacturers' protocols.

#### Northern-western blotting for miRNA

20 μg total RNA (add 20 μl DEPC H2O, 20 μl formamide to RNA sample and then heat RNA at 65°C for 10 min following chill on ice for 1 min) was separated on 12% polyacrylamide/8M urea gel (Amersham Pharmacia, Uppsala, Sweden) in a Protean II apparatus (BioRad, Hercules, CA, USA) (gel preheated to 55°C by electric current and water bath circulation before loading sample). 21nt RNAs are near the middle position between dye of bromophenol blue and xylene FF in such PAGE gel. Soak Hybond-N+ membrane (Amersham) in ddH2O for a few seconds and in transfer buffer (0.5X TBE) for 15 minutes, and soak 2 pieces of whatman paper in 0.5XTBE. Set up transfer as such From Bottom (anode) to top : whatman, membrane, gel, whatman, cathode plate. Separated RNA in gel was electro-blotted onto Hybond-N+ membrane (Amersham) by Trans-Blot SD electrophoretic transfer cell (BioRad) at 400mA for 1 hour. After UV cross-linking and air drying, blotted membrane was prehybridized with Ultrahyb-oligo hybridization buffer (Ambion) at 37∼42°C for 60 min, hybridized with Biotin-labeled antisense probe of the target miR372 and incubated at 40°C for overnight. The membrane was washed 2∼4 times at 40°C with 2× SSC and 0.5% SDS for 15 min and then western blotting with anti-Biotin.

#### Western blotting

The logarithmically growing cells were washed twice with ice-cold phosphate-buffered saline (PBS, Hyclone) and lysed in a RIPA lysis buffer. Cells lysates were centrifuged at 12,000g for 20 minutes at 4°C after sonication on ice, and the supernatant were separated. After being boiled for 5–10 minutes in the presence of 2-mercaptoethanol, samples containing cellulars proteins were separated on a 10% sodium dodecyl sulfate-polyacrylamide gel electrophoresis (SDS-PAGE) and transferred onto a nitrocellulose membrane, blocked in 10% dry milk-TBST (20 mM Tris-HCl [PH 7.6], 127 mM NaCl, 0.1% Tween 20) for 1 h at 37°C. Following three washes in Tris-HCl pH 7.5 with 0.1% Tween 20, the blots were incubated with 0.2 μg/ml of antibody(appropriate dilution) overnight at 4°C. Following three washes, membranes were then incubated with secondary antibody for 60 min at 37°C or 4°C overnight in TBST. Signals were visualized by ODYSSEY infrared imaging system.

#### Co-immunoprecipitation(IP)

Cells were lysed in 1 ml of the whole-cell extract buffer A (50 mM pH7.6 Tris-HCl, 150 mMNaCl, 1%NP40, 0.1 mMEDTA,1.0 mM DTT,0.2 mMPMSF, 0.1 mM Pepstatine,0.1 mM Leupeptine,0.1 mM Aproine). 500 μl cell lysates were used for immunoprecipitation with antibody. In brief, protein was pre-cleared with 30 μl protein G/A-plus agarose beads (Santa Cruz, Biotechnology, Inc., CA, USA) for 1 hour at 4°C and the supernatant was obtained after centrifugation (5,000 rpm) at 4°C. Precleared homogenates(supernatant) were incubated with 2 μg of antibody and/or normal mouse/rabbit IgG with rotation for 4 hours at 4°C. The immunoprecipitates were incubated with 30 μl protein G/A-plus agarose beads by rotation overnight at 4°C, and then centrifuged at 5000 rpm for 5 min at 4°C. The precipitates were washed five times for 10 min with beads wash solution (50 mM pH7.6 Tris-HCl,150 mMNaCl,0.1%NP-40,1 mM EDTA), resuspended in 60 μl 2×SDS-PAGE sample loading buffer and incubate for 5–10 min at 100°C. Western blotting was performed with related antibodies indicated in Figure.

#### RNA Immunoprecipitation(RIP)

Cells were lysed (15 min, 0°C) in 100 mM KCl, 5 mM MgCl_2_, 10 mM HEPES [pH 7.0], 0.5% NP40, 1 mM DTT, 100 units/ml RNase OUT (Invitrogen), 400 μM vanadyl-ribonucleoside complex and protease inhibitors (Roche), clarified and stored on at −80°C. Ribonucleoprotein particle-enriched lysates were incubated with protein A/G-plus agarose beads (Santa Cruz, Biotechnology, Inc.CA) together with the antibody or normal mouse or rabbit IgG for 4 hours at 4°C. Beads were subsequently washed four times with 50 mM Tris-HCl(pH 7.0), 150 mM NaCl, 1 mM MgCl_2_, and 0.05% NP-40, and twice after addition of 1M Urea. Immunoprecipitates(IPs) were digested with proteinase K and mRNAs were then isolated and purified, and RT-PCR was performed.

#### Chromatin immunoprecipitation (CHIP) assay

Cells were cross-linked with 1% (v/v) formaldehyde (Sigma) for 10 min at room temperature and stopped with 125 mm glycine for 5 min. Crossed-linked cells were washed with phosphate-buffered saline, resuspended in lysis buffer, and sonicated for 8–10 min in a SONICS VibraCell to generate DNA fragments with an average size of 500 bp. Chromatin extracts were diluted 5-fold with dilution buffer, pre-cleared with Protein-A/G-Sepharose beads, and immunoprecipitated with antibodies on Protein-A/G-Sepharose beads. After washing, elution and de-cross-linking, the ChIP DNA was detected by PCR.

#### Super-EMSA(Gel-shift)

Cells were washed and scraped in ice-cold PBS to prepare nuclei for electrophoretic gel mobility shift assay with the use of the gel shift assay system modified according to the manufacturer's instructions (Promega).

### Chromosome conformation capture (3C) -chromatin immunoprecipitation (ChIP) assay(ChIP-3C/ChIP-Loop assays)

Antibody-specific immunoprecipitated chromatin was obtained as described above for ChIP assays. Chromatin still bound to the antibody-Protein-A/G-Sepharose beads were resuspended in 500 μl of 1.2× restriction enzyme buffer at 37°C for 1 h. 7.5 μl of 20% SDS was added, the mixture was incubated for 1 h, followed by addition of 50 μl of 20% Triton X-100, incubated for an additional 1 h. Samples were then incubated with 400 units of selected restriction enzyme at 37°C overnight. After digestion, 40 μl of 20% SDS was added to the digested chromatin, and the mixture was incubated at 65°C for 10 min. 6.125 ml of 1.15× ligation buffer and 375 μl of 20% Triton X-100 was added, the mixture was incubated at 37°C for 1 h, and then 2000 units of T4 DNA ligase was added at 16°C for a 4-h incubation. Samples were then de-cross-linked at 65°C overnight followed by phenol-chloroform extraction and ethanol precipitation. After purification, the ChIP-3C material was detected for long range interaction with specific primers.

#### Cells proliferation CCK8 assay

Cells were synchronized in G0 phase by serum deprivation, then released from growth arrest by reexposure to serum, and grown in complete medium according to the manufacturer's instructions (Boshide, Wuhan, China).

#### Colony-formation efficiency assay

5 × 10^2^ cells were plated on a 10 cm dish, the 10 ml DMEM containing 10%FBS was added into each 10 cm dish of the three replicate. Then these dishes were incubated at 37°C in humidified incubator for 10 days. Cell colonies on the dishes were stained with 1 ml of 0. 5% Crystal Violet for more than 1 hour and the colonies were counted.

#### Xenograft transplantation *in vivo*

Four-weeks old male athymic Balb/C mice were purchased from Shi Laike Company (Shanghi, China) and maintained in the Tongji animal facilities approved by the China Association for Accreditation of Laboratory Animal Care. The athymic Balb/C mice were injected in the armpit area subcutaneously with Hep3B suspension of 1 × 10^8^ cells in 100 μl of phosphate buffered saline. The mice were observed four weeks, and then sacrificed to recover the tumors. The wet weight of each tumor was determined for each mouse. A portion of each tumor was fixed in 4% paraformaldehyde and embedded in paraffin for histological hematoxylin-eosin(HE) staining.

## RESULTS

### JMJD2A accelerates growth of liver cancer cells

**Figure 1:**
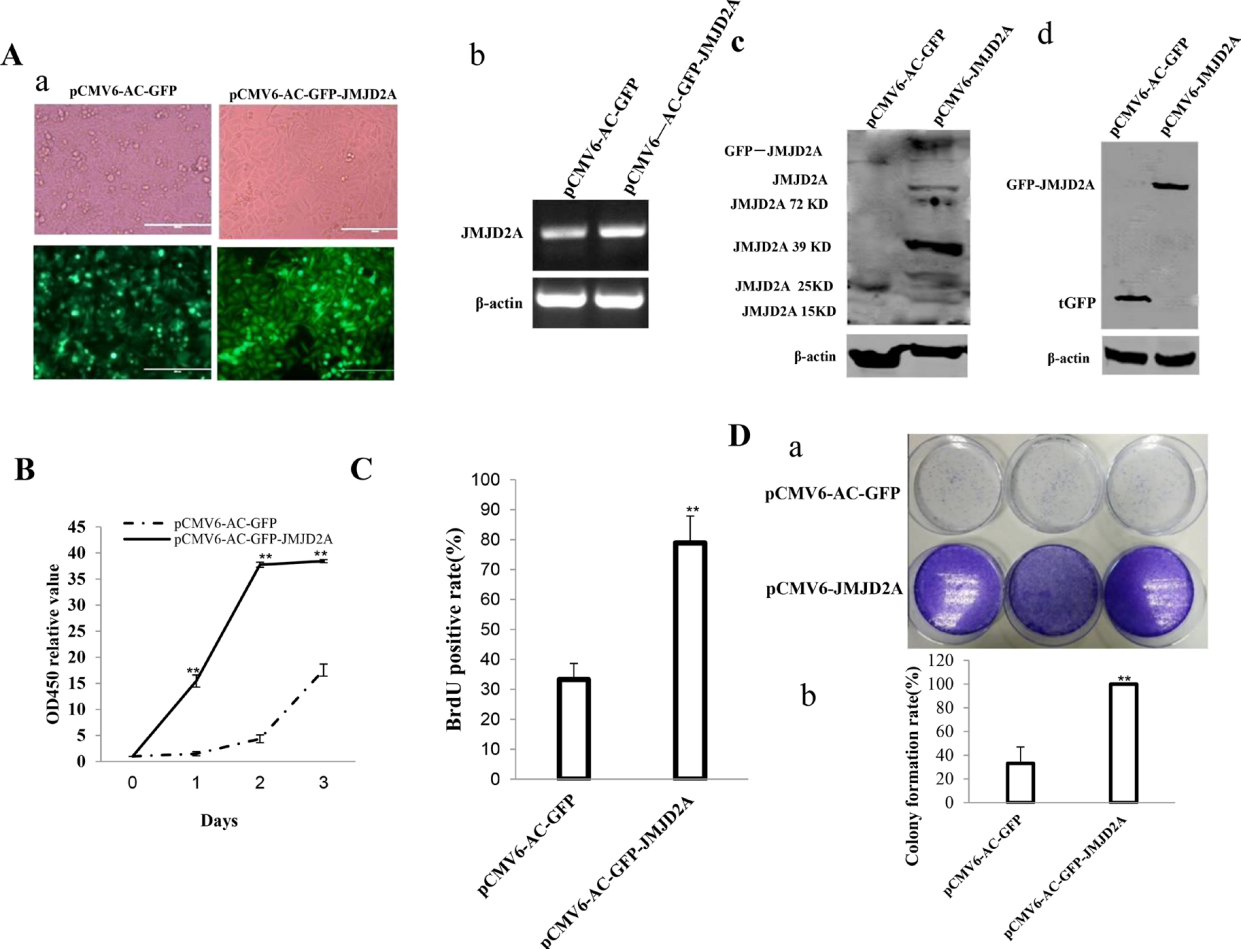
JMJD2A accelerates liver cancer cell growth *in vitro* (**Aa**) The photography of the Hep3B cell lines transfected with pCMV6-AC-GFP or pCMV6-AC-GFP-JMJD2A. (**Ab**) RT-PCR for JMJD2A cDNA in JMJD2A overexpressed control Hep3B stable cell lines;β-actin as internal control. (**Ac**) Western blotting with anti- JMJD2A in JMJD2A overexpressed control Hep3B stable cell lines; β-actin as internal control. (**Ad**) Western blotting with anti-tGFP in JMJD2A overexpressed control Hep3B stable cell lines; β-actin as internal control. (**B**) Cell proliferation assay was performed in 96-well format using the CCK8 cells proliferation kit to determine the cell viability as described by the manufacturer. Each sample was assayed in triplicates for 3 days consecutively. Cell growth curve was based on the corresponding the relative values of OD450 and each point represents the mean of three independent samples. Data are means of value from three independent experiments, bar ± SEM. **P < 0.01; *P < 0.05. (**C**) Cell BrdU assay. Data are means of value from three independent experiment, bar ± SEM. **P < 0.01; *P < 0.05. (**D**) (*upper*) The photography of colonies from the cell lines indicated in left. (*lower*) Cell plate colony formation ability assay. Data are means of value from three independent experiment, bar ± SEM. **P < 0.01; *P < 0.05.

**Figure 2:**
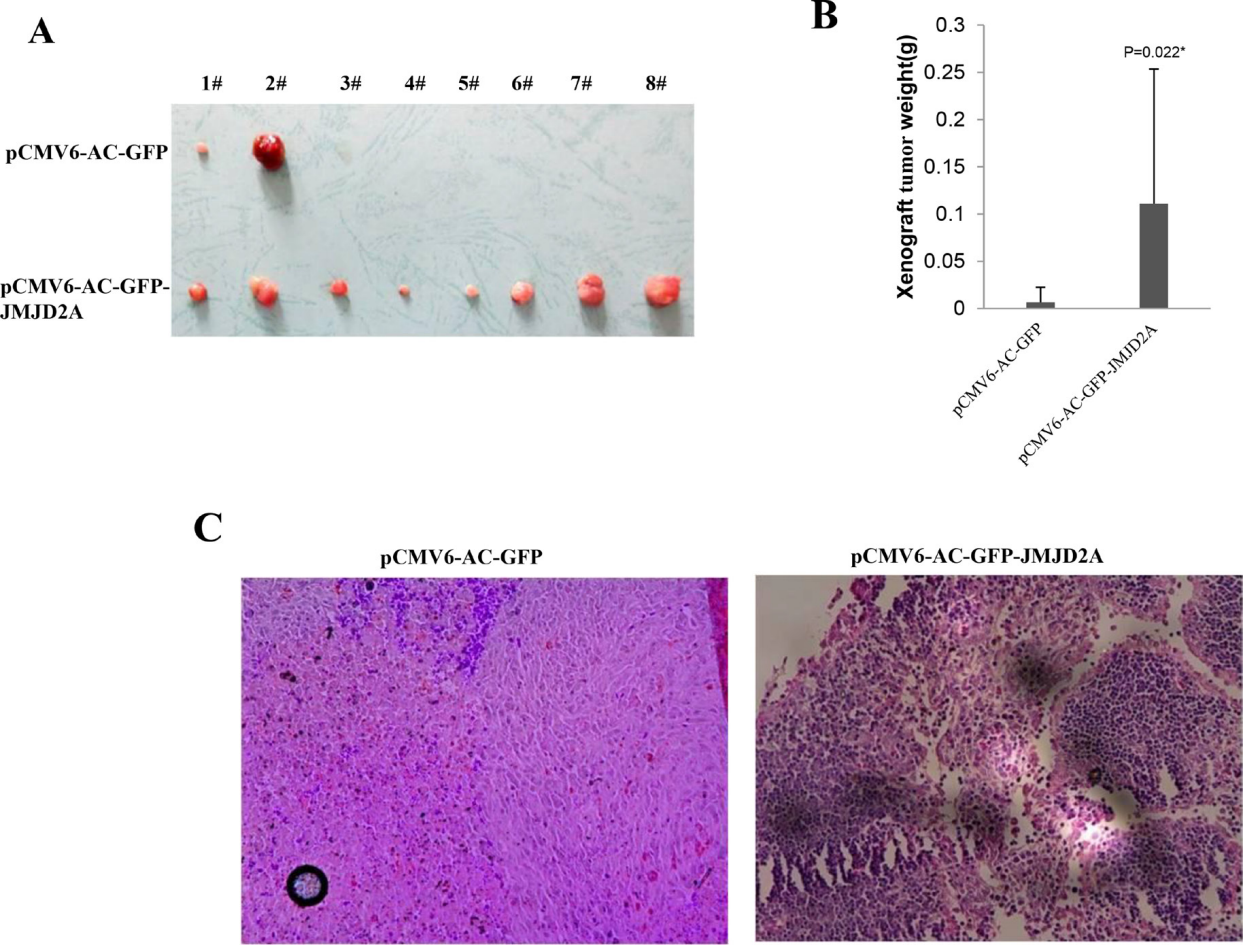
JMJD2A promotes liver cancer cell growth *in vivo* (**A**) The photography of xenograft tumors from Balb/C null mouse injected with Hep3B cells transfected with pCMV6-AC-GFP, pCMV6-AC-GFP-JMJD2A subcutaneously at armpit (**B**) The xenograft tumors weight(gram) in the two groups. Data were means of value from nine Balb/c mice, mean ± SEM, *n* = 8, *P < 0.05; **P < 0.01. Data were means of value from nine Balb/c mice, mean ± SEM, *n* = 8, *P < 0.05; **P < 0.01. (**C**) A portion of each xenograft tumor was fixed in 4% formaldehyde and embedded in paraffin, and the micrometers of sections (4 μm) were made for hematoxylin-eosin (HE) staining (original magnification×100).

To investigate whether JMJD2A promoted malignant growth of human liver cancer cell line Hep3B, we first screened two stable Hep3B cell lines transfectd with pCMV6-AC-GFP(GFP ctrl), pCMV6-AC-GFP-JMJD2A(JMJD2A) respectively. As shown in Figure 1Aa–1Ad, the expression of JMJD2A mRNA or protein was significantly increased in JMJD2A overexpressing Hep3B. As shown in Figure 1B, excessive JMJD2A significantly increased the growth of liver cancer cell Hep3B compared to the control group (*P* < 0.01). Furthermore, we performed colony formation assay and observed a significant increase in colony formation efficiency rate in excessive JMJD2A compared to control(100 ± 0%% versus 33.07 ± 13.98%, *P* = 0.00711 < 0.01) (Figure 1C). Moreover, JMJD2A overexpression significantly increased the BrdU positive rate compared to the control cells (33.25 ± 5.39% versus 78.91 ± 8.97%, *p* = 0.01477 < 0.05) (Figure 1D). To explore the effect of JMJD2A on liver cancer cells *in vivo*, the two stable Hep3B were injected subcutaneously into Balb/C mice. As shown in Figure 2A, 2B, when JMJD2A was overexpressed, the xenograft tumor weight increased approximately 16.419 folds compared to the corresponding control group (0.006728 ± 0.01585 grams versus 0.11047 ± 0.142461 grams, *P* = 0.0228 < 0.05). Moreover, compared to control, xenograft tumors contained more of poorly differentiated cells in JMJD2A overexpression group (Figure 2C). Taken together, these findings demonstrate that JMJD2A accelerates malignant progression of liver cancer cells.

### JMJD2A enhances miR372 expression epigenetically

**Figure 3:**
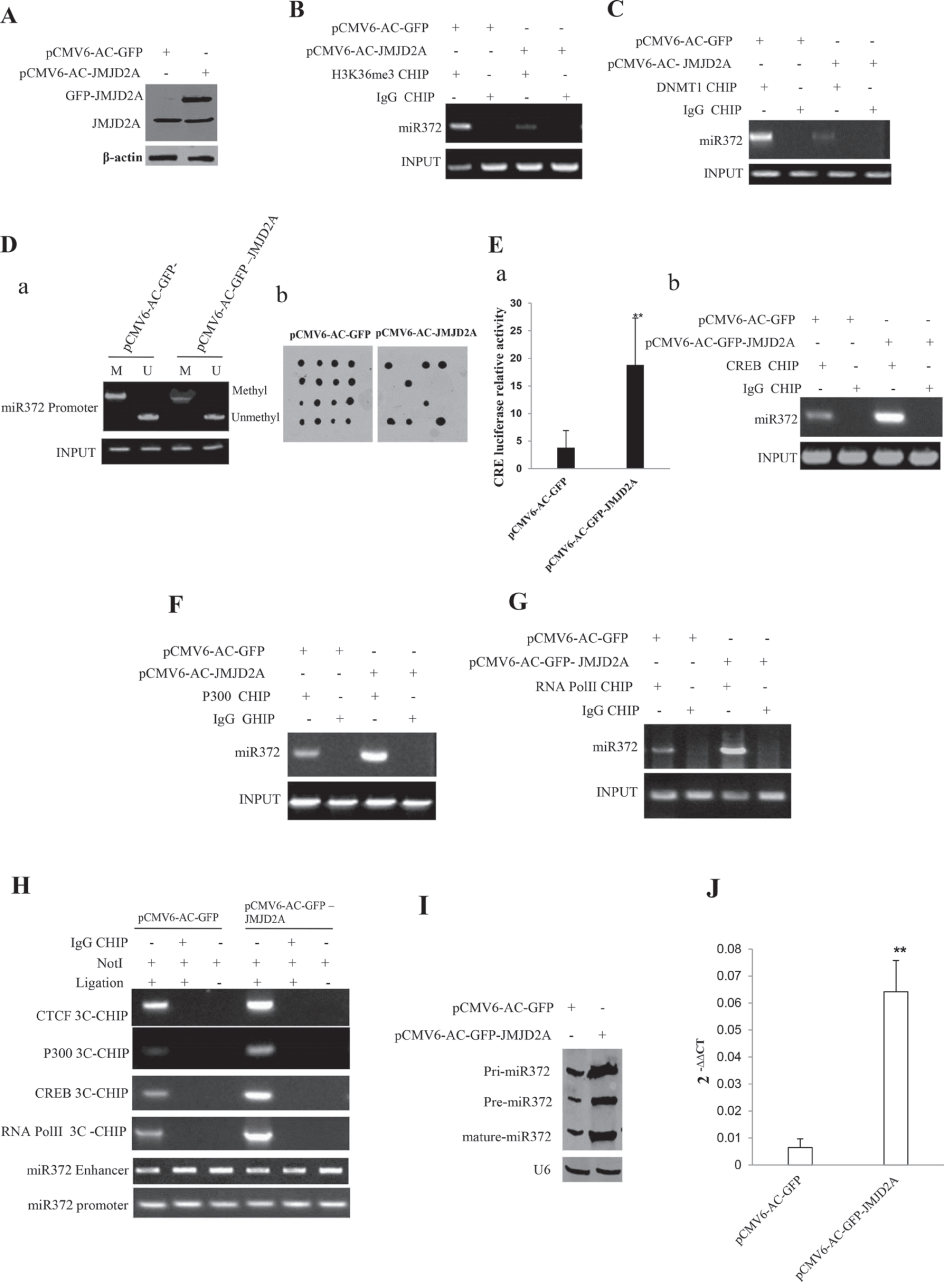
JMJD2A enhances miR372 expression epigenetically (**A**) Western blotting analysis with anti-JMJD2A in liver cancer cells Hep3B cell lines transfected with pCMV6-AC-GFP, pCMV6-AC-GFP-JMJD2A respectively. β-actin as internal control. (**B**) Chromatin Immunoprecipitation(CHIP) with anti-H3K9me3 followed by PCR with miR372 promoter primers in liver cancer cells Hep3B cell lines transfected with pCMV6-AC-GFP, pCMV6-AC-GFP-JMJD2A respectively. IgG CHIP as negative control. miR372 promoter as INPUT. (**C**) Chromatin Immunoprecipitation(CHIP) with anti-DNMT1 followed by PCR with miR372 promoter primers in liver cancer cells Hep3B cell lines transfected with pCMV6-AC-GFP, pCMV6-AC-GFP-JMJD2A respectively. IgG CHIP as negative control. miR372 promoter as INPUT. (**Da**) Methylation specific PCR (MSP) analysis for miR372 promoter region in Hep3B celllines transfected with pCMV6-AC-GFP, pCMV6-AC-JMJD2A, respectively. pw primer amplification as internal control. methyl DNA fragment is 170 bp.unmethyl DNA fragment is 130 bp. (**Db**) The dot blot analysis of miR372 promoter DNA methylation using specific biotin-DNA methylation probe. (**Ea**) CRE binding element luciferase activity assay. (**Eb**) Chromatin Immunoprecipitation(CHIP) with anti-CREB followed by PCR with miR372 promoter primers in liver cancer cells Hep3B cell lines transfected with pCMV6-AC-GFP, pCMV6-AC-GFP-JMJD2A respectively. IgG CHIP as negative control. miR372 promoter as INPUT. (**F**) Chromatin Immunoprecipitation(CHIP) with anti-P300 followed by PCR with miR372 promoter primers in liver cancer cells Hep3B cell lines transfected with pCMV6-AC-GFP, pCMV6-AC-GFP-JMJD2A respectively. IgG CHIP as negative control. miR372 promoter as INPUT). (**G**) Chromatin Immunoprecipitation(CHIP) with anti-RNAPolII followed by PCR with miR372 promoter primers in liver cancer cells Hep3B cell lines transfected with pCMV6-AC-GFP, pCMV6-AC-GFP-JMJD2A respectively. IgG CHIP as negative control. miR372 promoter as INPUT. (**H**) Chromosome conformation capture (3C)-chromatin immunoprecipitation (ChIP) with anti-P300,anti-RNA polII,anti-CREB in liver cancer cells Hep3B cell lines transfected with pCMV6-AC-GFP, pCMV6-AC-GFP-JMJD2A respectively. The chromatin is cross-linked, digested with restriction enzymes, and ligated under conditions that favor intramolecular ligation. Immediately after ligation, the chromatin is immunoprecipitated using an antibody (anti-P300,anti-RNA polII)against the protein of interest. Thereafter, the cross-links are reversed, and the DNA is purified further. The PCR anlysis is applied for detecting miR372 promoter-enhancer coupling product using miR372 promoter and enhancer primers. The miR372 promoter and enhancer as INPUT. F.Biotin-pre-miR372 pulldown followed by Western blotting with anti-Dicer, anti-ago2 Biotin as INPUT and β-actin as internal control. (**I**) Northern blotting analysis of miR372 in liver cancer cells transfected with pCMV6-AC-GFP, pCMV6-AC-GFP-JMJD2A. (**J**) The real-time PCR detection of mature miR372 in liver cancer cells transfected with pCMV6-AC-GFP, pCMV6-AC-GFP-JMJD2A respectively. Each value was presented as mean ± standard error of the mean (SEM). **P < 0.01.

Given our previous study showed JMJD2A is positively associated with miR372 in human liver cancer tissues, we consider whether JMJD2A enhances miR372 expression. As shown in Figure 3A, JMJD2A was overexpressed in Hep3B cell line transfected with pCMV6-AC-GFP-JMJD2A. In the JMJD2A overexpressed Hep3B cell lines, the JMJD2A inhibited the interplay between H3K36me3 and miR372 promoter (Figure 3B) and the interplay between DNMT1 and miR372 promoter (Figure 3C). JMJD2A inhibited the methylation of miR372 promoter region (Figure 3Da and 3Db). Moreover, JMJD2A enhanced the CRE element luciferase activity (Figure 3Ea) and the loading of CREB on the miR372 promoter region (Figure 3Eb). Furthermore, JMJD2A enhanced the the loading of P300 and RNApolII on the miR372 promoter region (Figure 3E, 3G). Intriguingly, JMJD2A promoted the formation of CTCF mediated promoter-enhancer DNA loop of miR372 and triggered CREB, P300, RNApolII into the DNA loop (Figure 3H). Ultimately, pri-miR372, pre-miR372 and mature miR372 were significantly increased in JMJD2A overexpressing Hep3B compared to control group (Figure 3I, 3J). Together, these observations suggest that JMJD2A promoted the expression and mature of miR372 epigenetically.

### miR372 triggers a novel transcript (JMJD2AΔ) of JMJD2A

**Figure 4:**
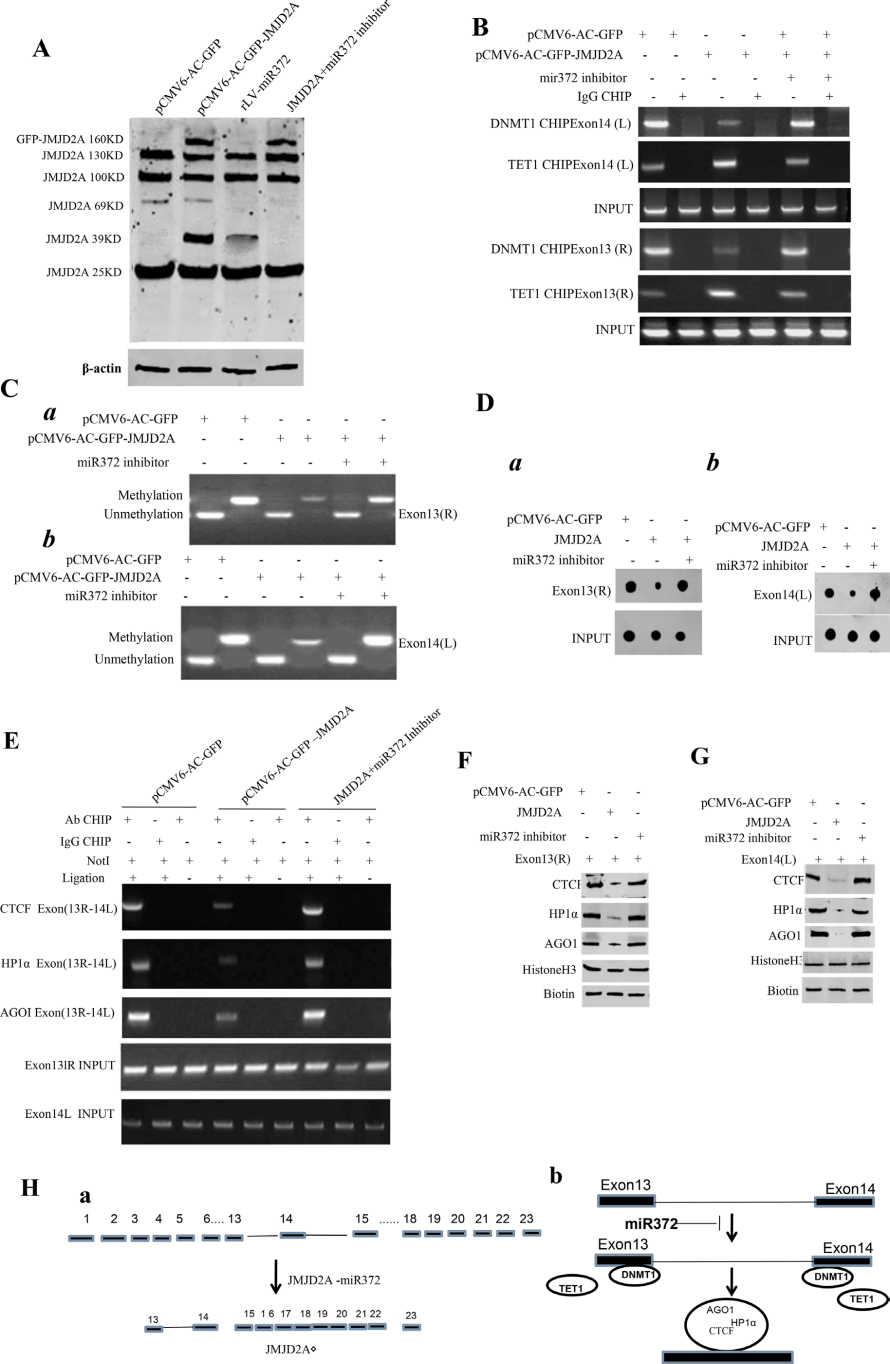
miR372 triggers a novel transcript(JMJD2AΔ) of JMJD2A (**A**) Western blotting analysis with anti-JMJD2A in liver cancer cells Hep3B cell lines transfected with pCMV6-AC-GFP, pCMV6-AC-GFP-JMJD2A,rLV-miR372, pCMV6-AC-GFP-JMJD2A plus miR372 inhibitor respectively. β-actin as internal control. (**B**) Chromatin Immunoprecipitation (CHIP) with anti-DNMT1 and anti-TET followed by PCR with JMJD2A exon13 or 14 primers in liver cancer cells Hep3B cell lines transfected with pCMV6-AC-GFP, pCMV6-AC-GFP-JMJD2A and pCMV6-AC-GFP-JMJD2A plus miR372 inhibitor respectively. IgG CHIP as negative control. JMJD2A exon13 or 14 as INPUT. (**C**) Methylation specific PCR (MSP) analysis for JMJD2A exon13 right region (a) or JMJD2A exon14 left region (b) in Hep3B cell lines transfected with pCMV6-AC-GFP, pCMV6-AC-JMJD2A and pCMV6-AC-GFP-JMJD2A plus miR372 inhibitor respectively .pw primer amplification as internal control. methyl DNA fragment is 150 bp.unmethyl DNA fragment is 120 bp. (**D**) The dot blot analysis for JMJD2A exon13 right region (a) or JMJD2A exon14 left region (b) DNA methylation using specific biotin-DNA methylation probe. (**E**) Chromosome conformation capture (3C)-chromatin immunoprecipitation (ChIP) with anti-CTCF,anti-HP1α,anti-AGOI in liver cancer cells Hep3B cell lines transfected with pCMV6-AC-GFP and pCMV6-AC-GFP-JMJD2A plus miR372 inhibitor respectively. The PCR anlysis is applied for detecting JMJD2A exon13(right)-exon14(left) coupling product using JMJD2A exon13 and exon 14 primers. The JMJD2A exon13 and exon 14 as INPUTI. (**F**) Biotin-JMJD2A exon13 DNA pulldown followed by Western blotting with anti-CTCF,anti-HP1α,anti-AGOI .Biotin as INPUT and Histone H3 as internal control. (**G**) Biotin-JMJD2A exon14 DNA pulldown followed by Western blotting with anti-CTCF,anti-HP1α,anti-AGOI .Biotin as INPUT and Histone H3 as internal control. (**Ha**) Informatics analysis :JMJD2A contains 23 exons(1–23 exon), while JMJD2AΔ contains only contain 7 exons(15–22). (**Hb**) The schematic illustrates a model of JMJD2A exon-intron editing blocked by miR372.

Given JMJD2A exists different transcripts, we wonder whether miR372 is involved in JMJD2A exon-intron editing. As shown in Figure 4A, a 39KD JMJD2A transcript, JMJD2AΔ, was significantly increased in JMJD2A or miR372 overexpressing Hep3B cell line. However, the JMJD2AΔ was disappeared in JMJD2A overexpressing plus miR372 knockdown Hep3B cell line. Moreover, JMJD2A decreased the loading of DNMT1 and increased the loading of TET1 on the left flank of 14th exon and on the right flank of 13th exon. However, miR372 inhibitor abrogated this JMJD2A action (Figure 4B). Therefore, JMJD2A decreased the DNA methylation on the left flank of 14th exon and on the on the right flank of 13th exon. However, miR372 inhibitor abrogated this JMJD2A action by MSP PCR experiment (Figure 4Ca, 4Cb) or Dot blot assay (Figure 4Da, 4Db). Furthermore, JMJD2A decreased intron DNA looping within the 13th exon-14th exon by CTCF-3C-CHIP and decreased the CTCF,AgoI, HP1α in the DNA looping. However, miR372 inhibitor abrogated this JMJD2A action by CHIP (Figure 4E). In assay *in vitro*, we found that JMJD2A decreased the interplay between CTCF, AgoI, HP1α and the probe of right flank of 13th exon. However, miR372 inhibitor abrogated this JMJD2A action by DNA pulldown (Figure 4F). And excessive JMJD2A decreased the interplay between CTCF, AgoI, HP1α and the probe of left flank of 14th exon. However, miR372 inhibitor abrogated this JMJD2A action by CHIP (Figure 4G). Informatics analysis suggests that JMJD2A contains 23 exons(1–23 exon), while JMJD2AΔ contains only contain 7 exons z(15–22) (Figure 4Ha). Therefore, we infer it is associated with exon-intron editing, e.g. 13th exon-introns-14th exon (Figure 4Hb). Taken together, miR372 blocks the editing of 13th exon-introns-14th exon of JMJD2A and a novel transcript(JMJD2AΔ) was formed in liver cancer cells.

### JMJD2A inhibits P21(WAF1/Cip1) via JMJD2AΔ

**Figure 5:**
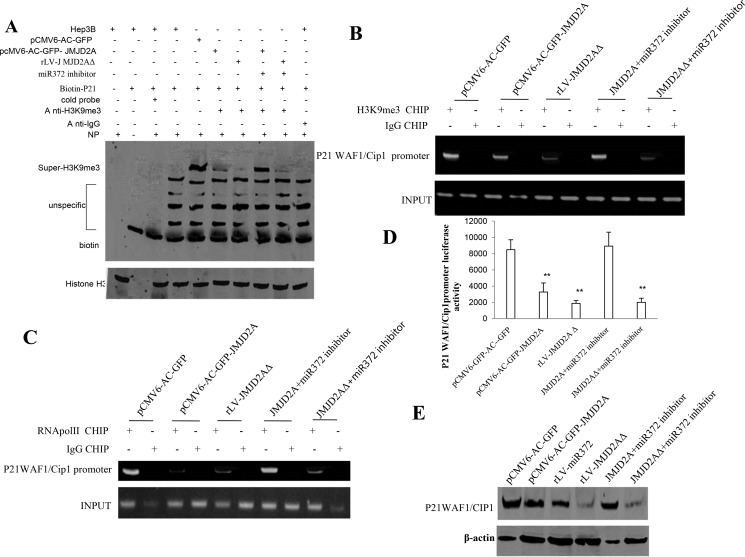
JMJD2A inhibits P21(WAF1/Cip1) via JMJD2AΔ (**A**) Super-EMSA(gel-shift) with biotin-P21WAF1/Cip1 promoter probe and anti-H3K9me3 antibody. The intensity of the band was examined by Western blotting with anti-Bioton.HistoneH3 as internal control. (**B**) Chromatin Immunoprecipitation(CHIP) with anti-H3K9me3 followed by PCR with P21 WAF1/Cip1 promoter primers in liver cancer cells Hep3B cell lines transfected with pCMV6-AC-GFP, pCMV6-AC-GFP-JMJD2A, rLV-JMJD2AΔ, pCMV6-AC-GFP-JMJD2A plus miR372 inhibitor, rLV-JMJD2AΔ plus miR372 inhibitor, respectively. IgG CHIP as negative control. P21 WAF1/Cip1 promoter as INPUT. (**C**) Chromatin Immunoprecipitation(CHIP) with anti-RNApolII followed by PCR with P21 WAF1/Cip1 promoter primers in liver cancer cells Hep3B cell lines transfected with pCMV6-AC-GFP, pCMV6-AC-GFP-JMJD2A, rLV-JMJD2AΔ, pCMV6-AC-GFP-JMJD2A plus miR372 inhibitor, rLV-JMJD2AΔ plus miR372 inhibitor, respectively. IgG CHIP as negative control. P21 WAF1/Cip1 promoter as INPUT. (**D**) The assay of P21 WAF1/Cip1 promoter luciferase activety. Each value was presented as mean ± standard error of the mean (SEM).**P < 0.01. (**E**) Western blotting analysis with anti- P21WAF1/CIP1 in liver cancer cells Hep3B cell lines transfected with pCMV6-AC-GFP, pCMV6-AC-GFP-JMJD2A, rLV-miR372, rLV-JMJD2AΔ, pCMV6-AC-GFP-JMJD2A plus miR372 inhibitor, rLV-JMJD2AΔ plus miR372 inhibitor, respectively. β-actin as internal control. Antisense-mature miR372 as miR372 inhibitor.

To identify whether JMJD2AΔ could regulate oncogenic function of JMJD2A, we considered to select target gene of JMJD2A in Hep3B cell lines, e.g. P21(WAF1/Cip1). We first analysed the P21(WAF1/Cip1) in groups including pCMV6-AC-GFP, pCMV6-AC-GFP-JMJD2A, rLV-miR372, rLV--JMJD2AΔ, pCMV6-AC-GFP-JMJD2A plus miR372 inhibitor, rLV-JMJD2AΔ plus miR372 inhibitor. The results show that the H3K9me3 binding to P21(WAF1/Cip1) promoter probe was significantly reduced in pCMV6-AC-GFP-JMJD2A, rLV--JMJD2AΔ, and rLV-JMJD2AΔ plus miR372 inhibitor compared to control. However, The H3K9me3 binding to P21(WAF1/Cip1) promoter DNA probe was significantly not altered in pCMV6-AC-GFP-JMJD2A plus miR372 inhibitor compared to control (Figure 5A). Moreover, the H3K9me3 loading on P21(WAF1/Cip1) promoter region was significantly reduced in pCMV6-AC-GFP-JMJD2A, rLV--JMJD2AΔ, and rLV -JMJD2AΔ plus miR372 inhibitor compared to control. However, the H3K9me3 loading on P21(WAF1/Cip1) promoter region was significantly not altered in pCMV6-AC-GFP-JMJD2A plus miR372 inhibitor compared to control (Figure 5B). Therefore, the RNA polII loading to P21(WAF1/Cip1) promoter region was significantly decreased in pCMV6-AC-GFP-JMJD2A,pCMV-miR372, rLV -JMJD2AΔ, and rLV-JMJD2AΔ plus miR372 inhibitor compared to control. However, The RNApolII binding to P21(WAF1/Cip1) promoter DNA probe was significantly not altered in pCMV6-AC-GFP-JMJD2A plus miR372 inhibitor compared to control (Figure 5C). Furthermore, the P21(WAF1/Cip1) promoter luciferase activity was significantly decreased in pCMV6-AC-GFP-JMJD2A, pCMV-miR372, rLV-JMJD2AΔ, and rLV-JMJD2AΔ plus miR372 inhibitor compared to control. However, the P21(WAF1/Cip1) promoter luciferase activity was significantly not altered in pCMV6-AC-GFP-JMJD2A plus miR372 inhibitor compared to control (Figure 5D). Ultimately, P21(WAF1/Cip1) was significantly reduced in pCMV6-AC-GFP-JMJD2A, rLV-miR372, rLV-JMJD2AΔ, and rLV -JMJD2AΔ plus miR372 inhibitor compared to control. However, the expression of P21(WAF1/Cip1) were significantly not altered in pCMV6-AC-GFP-JMJD2A plus miR372 inhibitor compared to control (Figure 5E). Taken together, these observations suggest JMJD2A inhibited P21(WAF1/Cip1) in cell cycle progress through JMJD2AΔ dependent on miR372.

### JMJD2A promotes Pim1 via suppressing P21(WAF1/Cip1) dependent on JMJD2AΔ

**Figure 6:**
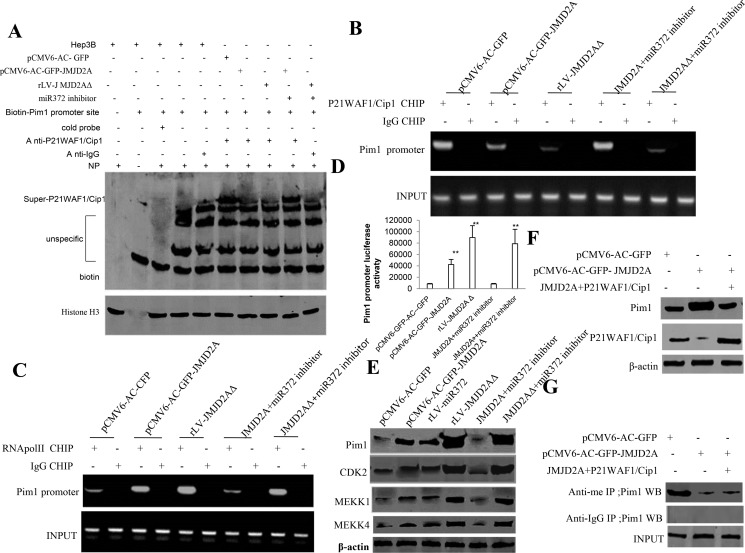
JMJD2A promotes Pim1 through suppressing P21(WAF1/Cip1) (**A**) Super-EMSA(gel-shift) with biotin-Pim1 promoter probe and anti- P21(WAF1/Cip1) antibody. The intensity of the band was examined by Western blotting with anti-Biotin.HistoneH3 as internal control. (**B**) Chromatin Immunoprecipitation(CHIP) with anti- P21(WAF1/Cip1) followed by PCR with Pim1 promoter primers in liver cancer cells Hep3B cell lines transfected with pCMV6-AC-GFP, pCMV6-AC-GFP-JMJD2A, rLV-JMJD2AΔ, pCMV6-AC-GFP-JMJD2A plus miR372 inhibitor, rLV-JMJD2AΔ plus miR372 inhibitor, respectively. IgG CHIP as negative control. Pim1 promoter as INPUT. (**C**) Chromatin Immunoprecipitation(CHIP) with anti-RNApolII followed by PCR with Pim1 promoter primers in liver cancer cells Hep3B cell lines transfected with pCMV6-AC-GFP, pCMV6-AC-GFP-JMJD2A, rLV-JMJD2AΔ, pCMV6-AC-GFP-JMJD2A plus miR372 inhibitor, rLV-JMJD2AΔ plus miR372 inhibitor, respectively. IgG CHIP as negative control. Pim1 WAF1/Cip1 promoter as INPUT. Antisense-mature miR372 as miR372 inhibitor. (**D**) The assay of Pim1 promoter luciferase activety. Each value was presented as mean ± standard error of the mean (SEM).**P < 0.01. (**E**) Western blotting analysis with anti- anti-Pim1, anti-CDK2, anti-MEKK1, anti-MEKK4 in liver cancer cells Hep3B cell lines transfected with pCMV6-AC-GFP, pCMV6-AC-GFP-JMJD2A, rLV-miR372, rLV-JMJD2AΔ, pCMV6-AC-GFP-JMJD2A plus miR372 inhibitor, rLV-JMJD2AΔ plus miR372 inhibitor, respectively. β-actin as internal control. (**F**) Western blotting analysis with anti-Pim1,anti-P21(WAF1/Cip1) in liver cancer cells Hep3B cell lines transfected with pCMV6-AC-GFP, pCMV6-AC-GFP-JMJD2A, pCMV6-AC-GFP-JMJD2A plus pcDNA3.1-P21(WAF1/Cip1), respectively. β-actin as internal control. (**G**) The assay of Pim1 methylation through Co-Immunoprecipitation(IP) with anti-me followed by Western blotting with anti-Pim1 in liver cancer cells Hep3B cell lines transfected with pCMV6-AC-GFP, pCMV6-AC-GFP-JMJD2A, pCMV6-AC-GFP-JMJD2A plus pcDNA3.1-P21(WAF1/Cip1), respectively. IgG IP as negative control. INPUT refers to Western blotting with anti-pim1.

To further identify whether JMJD2AΔ could regulate JMJD2A oncogenic function, we considered to select target gene of JMJD2A in Hep3B cell lines, e.g. Pim1. Our results show that the P21(WAF1/Cip1) binding to Pim1 promoter DNA probe was significantly reduced in pCMV6-AC-GFP-JMJD2A, rLV-JMJD2AΔ, and rLV-JMJD2AΔ plus miR372 inhibitor compared to control. However, The P21(WAF1/Cip1) binding to Pim1 promoter probe was significantly not altered in pCMV6-AC-GFP-JMJD2A plus miR372 inhibitor compared to control (Figure 6A). Moreover, the P21(WAF1/Cip1) loading on Pim1 promoter region was significantly reduced in pCMV6-AC-GFP-JMJD2A, rLV--JMJD2AΔ, and rLV-JMJD2AΔ plus miR372 inhibitor compared to control. However, The P21(WAF1/Cip1) loading on Pim1 promoter region was significantly not altered in pCMV6-AC-GFP-JMJD2A plus miR372 inhibitor compared to control (Figure 6B). Therefore, the RNA polII loading to Pim1 promoter region was significantly increased in pCMV6-AC-GFP-JMJD2A, rLV--JMJD2AΔ, and rLV-JMJD2AΔ plus miR372 inhibitor compared to control. However, The RNA pol II binding to pim1 promoter was significantly not altered in pCMV6-AC-GFP-JMJD2A plus miR372 inhibitor compared to control (Figure 6C). Furthermore, the Pim1 promoter luciferase activity was significantly increased in pCMV6-AC-GFP-JMJD2A, rLV-JMJD2AΔ and rLV-JMJD2AΔ plus miR372 inhibitor compared to control. However, the Pim1 promoter luciferase activity was significantly not altered in pCMV6-AC-GFP-JMJD2A plus miR372 inhibitor compared to control (Figure 6D). Finally, Pim1, CDK2 MEKK1, MKKK4 were significantly increased in pCMV6-AC-GFP-JMJD2A, rLV-miR372, rLV-JMJD2AΔ and rLV-JMJD2AΔ plus miR372 inhibitor compared to control. However, the expression of Pim1,CDK2 MEKK1, MEKK4 were significantly not altered in pCMV6-AC-GFP-JMJD2A plus miR372 inhibitor compared to control (Figure 6E). Strikingly, Pim1 were significantly increased in pCMV6-AC-GFP-JMJD2A compared to control. However, the expression of Pim1 was significantly not altered in pCMV6-AC-GFP-JMJD2A plus pcDNA3.1-P21(WAF1/Cip1) compared to control (Figure 6F). Of significance, the methylation of Pim1 were significantly reduced in pCMV6-AC-GFP-JMJD2A compared to control. However, the expression of methylation of Pim1 was significantly not altered in pCMV6-AC-GFP-JMJD2A plus pcDNA3.1-P21(WAF1/Cip1) compared to control (Figure 6G). Taken together, these observations suggest JMJD2A enhanced Pim1 by inhibiting P21(WAF1/Cip1) through JMJD2AΔ dependent on miR372.

### JMJD2A promotes cell cycle progression via Pim1-ppRB1-CDK2-CycinE-C-myc pathway

**Figure 7:**
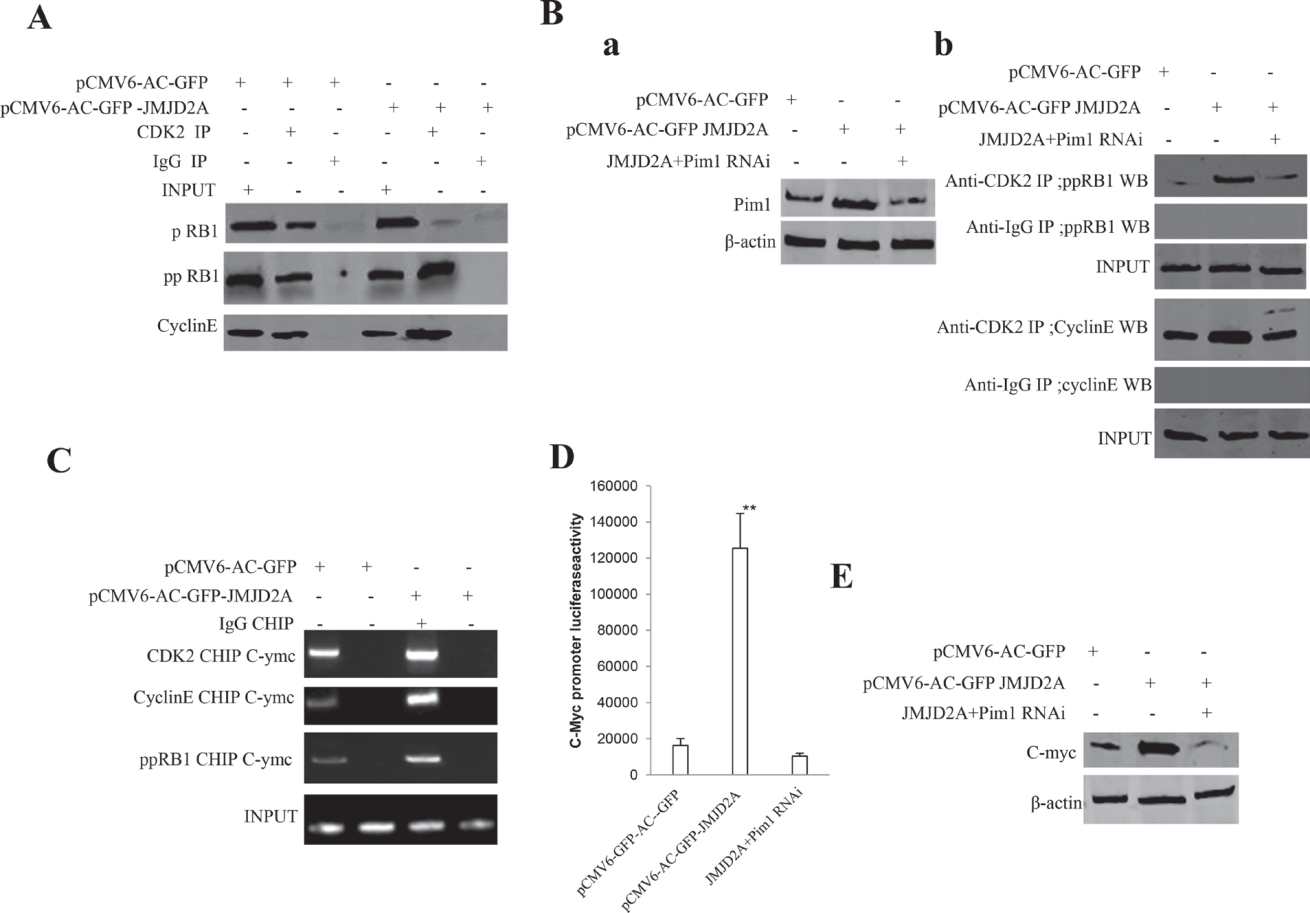
JMJD2A promotes C-myc expression by Pim1-CDK2-CyclinE-pRB axis (**A**) Co-Immunoprecipitation(IP) with anti-CDK2 followed by Western blotting with anti-CyclinE and anti-pRB1 in the Hep3B cell lines transfected with pCMV6-AC-GFP, pCMV6-AC-JMJD2A. IgG IP as negative control. INPUT refers to Western blotting with anti-pRB and anti-CyclinE. (**Ba**) Western blotting analysis with anti-Pim1 in liver cancer cells Hep3B cell lines transfected with pCMV6-AC-GFP, pCMV6-AC-GFP-JMJD2A, pCMV6-AC-GFP-JMJD2A plus pGFP-V-RS-Pim1, respectively. β-actin as internal control. (**Bb**) Co-Immunoprecipitation(IP) with anti-CDK2 followed by Western blotting with anti-CyclinE and anti-pRB1 in the Hep3B cell lines transfected with pCMV6-AC-GFP, pCMV6-AC-JMJD2A, and pCMV6-AC-JMJD2A plus pGFP-V-RS-pim1. IgG IP as negative control. INPUT refers to Western blotting with anti-pRB and anti-CyclinE. (**C**) Chromatin Immunoprecipitation(CHIP) with anti- CDK2 and anti-CyclinE followed by PCR with C-myc promoter primers in liver cancer cells Hep3B cell lines transfected with pCMV6-AC-GFP, pCMV6-AC-GFP-JMJD2A, respectively. IgG CHIP as negative control. C-myc1 promoter as INPUT. (**D**) The assay of C-myc promoter luciferase activety. Each value was presented as mean ± standard error of the mean (SEM).**P < 0.01. (**E**) Western blotting analysis with anti-C-myc in liver cancer cells Hep3B cell lines transfected with pCMV6-AC-GFP, pCMV6-AC-GFP-JMJD2A, pCMV6-AC-GFP-JMJD2A plus pGFP-V-RS-Pim1, respectively. β-actin as internal control.

Given that JMJD2A increased expression of Pim1 and decreased the methylation of Pim1 by inhibiting P21(WAF1/Cip1), we consider whether excessive JMJD2A may destroy the regulation and control of cell cycle proteins. As shown in Figure 7A, the interplay between CDK2 and CyclinE, CDK2 and ppRB1 was enhanced and the interplay between CDK2 and pRB1 was reduced in JMJD2A overexpressing Hep3B. However, Pim1 depletion fully abrogated the action of excessive JMJD2A that altered the interplay between CDK2 and CyclinE, CDK2 and ppRB1 and between CDK2 and pRB1 in Hep3B (Figure 7Ba, 7Bb). Moreover, the CDK2, CyclinE, and ppRB1 loading to C-myc promoter region was significantly increased in excessive JMJD2A group compared to control (Figure 7C). Furthermore, the C-myc promoter luciferase activity was significantly increased in Hep3B transfected with pCMV6-AC-GFP-JMJD2A compared to control, however, Pim1 knockdown abrogated this action of JMJD2A (Figure 7D). Ultimately, C-myc was significantly reduced in excessive JMJD2A group compared to control. However, the expression of C-myc were significantly not altered in Hep3B transfected with pCMV6-AC-GFP-JMJD2A plus pGFP-V-RS-Pim1 compared to control (Figure 7E). Taken together, these observations suggest JMJD2A promotes cell cycle progression via Pim1-ppRB1-CDK2-CycinE-C-myc pathway.

### Both Pim1 and P21(WAF1/Cip1) determine JMJD2A oncogenic function

**Figure 8:**
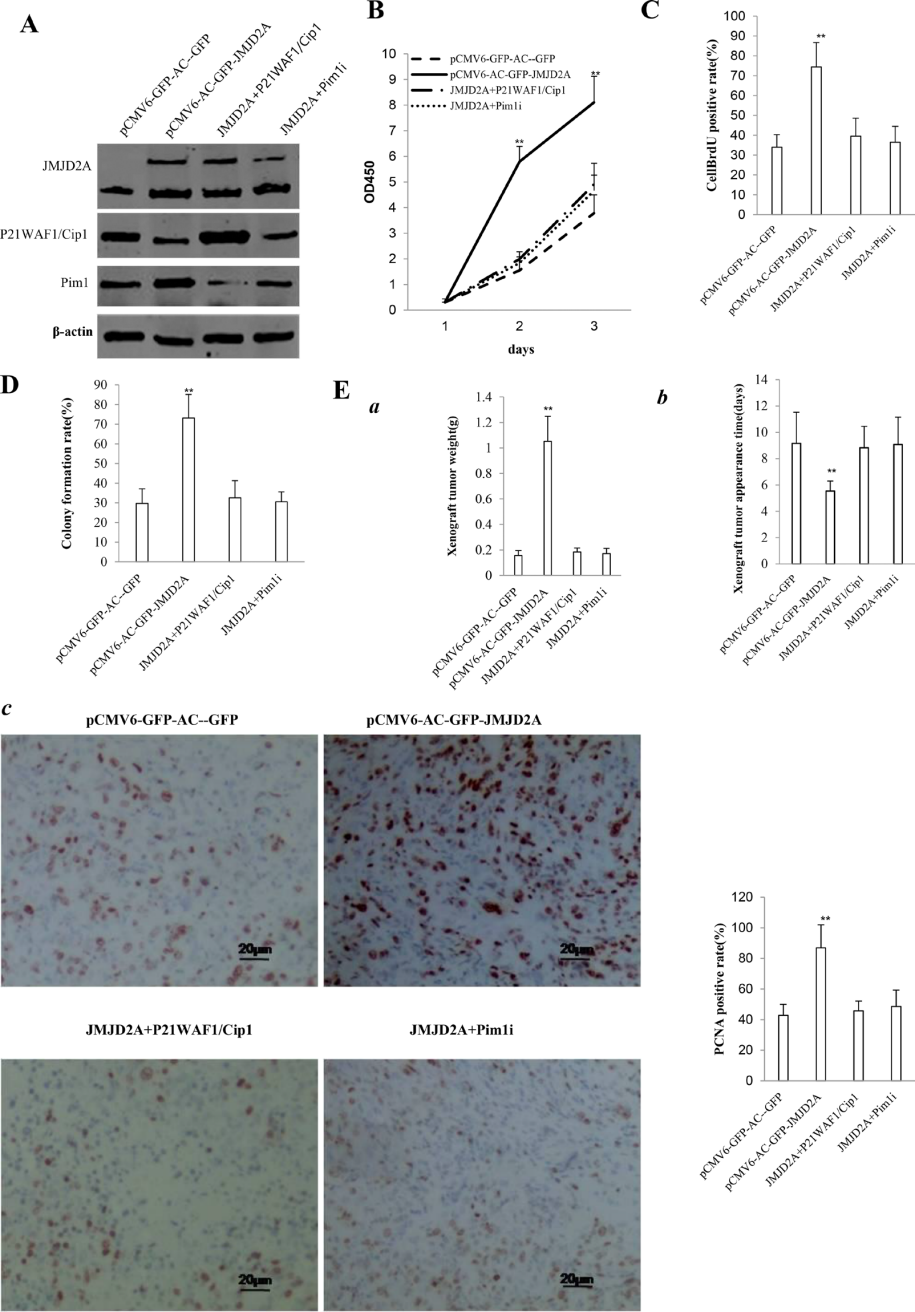
The rescued experiment of carcinogenesis effect of the JMJD2A in Hep3B cell lines transfected with pCMV6-AC-GFP, pCMV6-AC-JMJD2A (GFP-JMJD2A), pCMV6-AC-GFP-JMJD2A plus pcDNA3.1-P21WAF1/Cip1, pCMV6-AC-JMJD2A puls pGFP-V-RS-Pim1 (**A**) Western blotting analysis with anti-JMJD2A, and anti-P21WAF1/Cip1 and anti-Pim1. β-actin served as internal control. (**B**) Cells growth assay using CCK8. Each value was presented as mean ± standard error of the mean (SEM).**P < 0.01. (**C**) Cell BrdU staining assay. Each value was presented as mean ± standard error of the mean (SEM).**P < 0.01. (**D**) Cells soft agar colony formation assay. (**E**) Tumorigenesis test *in vivo* (a) The wet weight of each tumor was determined for each mouse. Each value was presented as mean ± standard error of the mean (SEM).**P < 0.01. (b) The appearance time of each tumor was determined for each mouse. Each value was presented as mean ± standard error of the mean (SEM).**P < 0.01. (c) A portion of each tumor was fixed in 4% paraformaldehyde and embedded in paraffin for PCNA staining (DAB stainning, original magnification×100).

To address whether JMJD2A oncogenic function is associated with Pim1 and P21(WAF1/Cip1), we constructed four stable Hep3B lines(pCMV6-AC-GFP, pCMV6-AC-GFP-JMJD2A, pCMV6-AC-GFP-JMJD2A plus pcDNA3.1 -P21(WAF1/Cip1), pCMV6-AC-GFP-JMJD2A plus pGFP-V-RS—Pim1). As shown in Figure 8A, JMJD2A was overexpressed in groups of pCMV6-AC-GFP-JMJD2A, pCMV6-AC-GFP-JMJD2A plus pcDNA3.1-P21(WAF1/Cip1), pCMV6-AC- GFP-JMJD2A plus pGFP-V-RS-Pim1 respectively. P21(WAF1/Cip1) was overexpressed in groups of pCMV6-AC-GFP-JMJD2A plus pcDNA3.1 -P21(WAF1/Cip1) and decreased in groups of pCMV6-AC-GFP-JMJD2A, pCMV6-AC-GFP-JMJD2A plus pGFP-V-RS-Pim1 respectively. Pim1 was knocked down in groups of pCMV6-AC-GFP-JMJD2A plus pGFP-V-RS-Pim1 and was enhanced in groups of pCMV6-AC-GFP-JMJD2A. Next, we detected the cell proliferation. As shown in Figure 8B, excessive JMJD2A significantly increased the growth of liver cancer cell Hep3B compared to the control cells (*P*< 0.01). However, both JMJD2A plus P21WAF1/Cip1 and JMJD2A plus Pim1RNAi did significantly not alter the growth of liver cancer cells compared to (*P* > 0.05). Moreover, excessive JMJD2A significantly increased the BrdU positive rate compared to the control cells (74.41 ± 12.29% versus 34.01. ± 6.32%, *p* = 0.003965 < 0.01). However, both JMJD2A plus P21WAF1/Cip1 and JMJD2A plus Pim1RNAi did significantly not alter the BrdU positive rate of liver cancer cells (39.52 ± 9.08%, 36.46 ± 8.02% versus34.01 ± 6.32%, *p* = 0.16246 > 0.05, *P* = 0.0683 > 0.05, respectively) (Figure 8C). We Further performed colony formation assay and observed a significant increase in colony formation efficiency rate in excessive JMJD2A group (73.17 ± 11.94% vs 29.73 ± 7.44%, *P* = 0.01566 < 0.05). However, both JMJD2A plus P21WAF1/Cip1 and JMJD2A plus Pim1RNAi did significantly not alter the colony formation rate of liver cancer cells (32.63 ± 8.66%, 30.64 ± 5.01% vs 29.73 ± 7.44%, *P* = 0.2027 > 0.05, 0.4544 > 0.05, respectively) (Figure 8D). Furthermore, the three stable Hep3B cell lines were injected subcutaneously into Balb/C mice. As shown in Figure 8E(a), when JMJD2A was overexpressed, the average xenograft tumor weight increased approximately 6.75 folds compared to the corresponding control group (1.05143 ± 0.19659 grams versus 0.15571 ± 0.03952 grams, *P* = 0.0000185 < 0.01). However, both JMJD2A plus P21WAF1/Cip1 and JMJD2A plus Pim1RNAi did significantly not alter the xenograft tumor weight (0.1829 ± 0.03251 gram,0.1714 ± 0.041 grams versus 0.15571 ± 0.03952 grams, *p* = 0.16 > 0.05, *P* = 0.2437 > 0.05, respectively). On the other hand, when JMJD2A was overexpressed, the average xenograft tumor appearance time was decreased compared to the corresponding control group (5.54286 ± 0.75687 days versus 9.15857 ± 2.36918 days, *P* = 0.0031 < 0.01). However, both JMJD2A plus P21WAF1/Cip1 and JMJD2A plus Pim1RNAi did significantly not alter the xenograft tumor appearance (8.82857 ± 1.62349 days, 9.07143 ± 2.07743 days versus 9.15857 ± 2.36918 days, *p* = 0.4016 > 0.05, *P* = 0.4735 > 0.05, respectively) (Figure 8Eb). Moreover, the expression of PCNA was significantly higher in JMJD2A overexpressing xenograft tumors compared to the control group (86.92 ± 15.01% versus 42.81 ± 7.13%, *P* = 0.01711 < 0.05). However, both JMJD2A plus P21WAF1/Cip1 and JMJD2A plus Pim1RNAi did significantly not alter the PCNA positive rate of liver cancer cells (45.7 ± 6.415%, 48.62 ± 10.68% versus 42.81 ± 7.13%, *P* = 0.3735 > 0.05, *P* = 0.3127 > 0.05) (Figure 8Ec). It suggested that JMJD2A promoted cell growth, colony formation ability and cell growth *in vivo*. However, both JMJD2A plus P21WAF1/Cip1 and JMJD2A plus Pim1RNAi abrogated the JMJD2A action. Taken together, both Pim1 and P21(WAF1/Cip1) determine oncogenic function of JMJD2A in liver cancer cells.

## DISCUSSION

**Figure 9:**
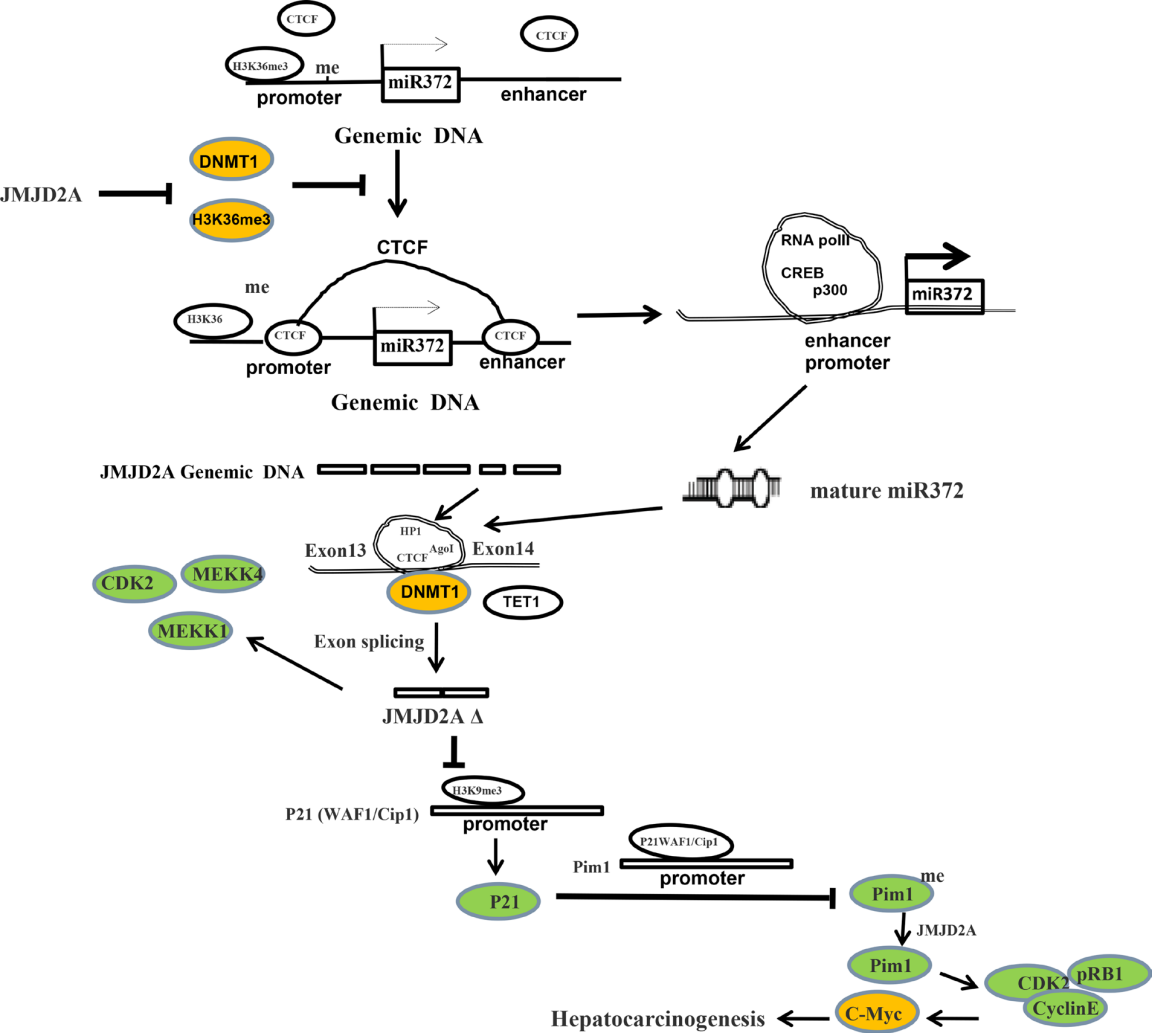
The schematic illustrates a model of JMJD2A promotes liver cancer cell growth through up-regulating miR372 JMJD2A accelerates malignant progression of liver cancer cells *in vitro* and *in vivo*. Mechanistically, JMJD2A promoted the expression and mature of miR372 epigenetically. Thereby, miR372 blocks the editing of 13th exon-introns-14th exon and forms a novel transcript (JMJD2AΔ) of JMJD2A. JMJD2A inhibited P21(WAF1/Cip1) expression by decreasing H3K9me3 dependent on JMJD2AΔ. Moreover, JMJD2A could enhance Pim1 transcription by suppressing P21(WAF1/Cip1). Furthermore, through increasing Pim1, JMJD2A could facilitate the interaction among pRB, CDK2 and CyclinE which prompts the transcription and translation of oncogenic C-myc. Strikingly, JMJD2A may trigger the demethylation of Pim1. Furthermore, Pim1 knockdown and P21(WAF1/Cip1) overexpression fully abrogated the oncogenic function of JMJD2A. JMJD2A promote liver cancer cell cycle progress through miR372-JMJD2AΔ- P21WAF1 /Cip1-Pim1- pRB-CDK2- CyclinE-C-myc axis.

Dysregulation of histone lysine methyltransferases is associated with carcinogenesis. Our studies indicated the effects of demethylase JMJD2A during hepatocarcinogenesis. Herein, our results demonstrate JMJD2A accelerates malignant progression of liver cancer cells. Mechanistically, JMJD2A promoted the expression and mature of miR372 epigenetically. Therefore, miR372 blocks the editing of 13th exon-introns-14th exon ofJMJD2A and prompts to form a novel transcript (JMJD2AΔ) of JMJD2A. Strikingly, JMJD2A inhibited P21(WAF1/Cip1) expression by decreasing H3K9me3 dependent on JMJD2AΔ. Moreover, JMJD2A could enhance Pim1 transcription by suppressing P21(WAF1/Cip1). Furthermore, through increasing Pim1, JMJD2A could facilitate the interaction among pRB, CDK2 and CyclinE which prompts the transcription and translation of oncogenic C-myc. Strikingly, JMJD2A may trigger the demethylation of Pim1. Of significance, Pim1 knockdown and P21(WAF1/Cip1) overexpression fully abrogated the oncogenic function of JMJD2A. Our observations suggest that JMJD2A promotes liver cancer cell cycle progress through JMJD2AΔ-P21WAF1/Cip1-Pim1- pRB-CDK2-CyclinE- C-myc axis dependent on miR372. (Figure 9).

To date, accumulating evidence indicates that JMJD2A results in a critical role in carcinogenesis and cancer metastasis. Excessive JMJD2A produces strong oncogenic functions [31]. JMJD2A is involved in the regulation of cancer cell cycle [32]. In particular, JMJD2A induces site-specific copy gain and amplification in cancer cancer [33, 34]. Herein, the functions of JMJD2A promotion of liver cancer stem cell growth is explained by results from two parallel sets of experiments: (1) JMJD2A is overexpressed and associated with miR372, P21(WAF1/Cip1), Pim1 in human liver cancer tissues; (2) JMJD2A accelerates malignant progression of liver cancer cells *in vitro* and *in vivo*.

Of significance, our observations demonstrated that miR372 influences on JMJD2A functions in liver cancer cells. The increase of miR372 may partly decide the JMJD2A-medicated promotion of liver cancer cell growth. Our findings in this study provide novel evidence for an active role of miR372 in this action. This assertion is based on several observations: (1) JMJD2A enhances miR372 expression epigenetically. (2) miR372 prompts s a novel transcript(JMJD2AΔ) of JMJD2A, a 39KD JMJD2A transcript (3) miR372 blocks the editing of 13th exon-introns-14th exon of JMJD2A.

Strikingly, our data suggest that miR372 influence on the editing of JMJD2A, involving in CTCF, DNMT1, HP1α. In particular, a novel transcript, JMJD2AΔ was formed. Increase of JMJD2AΔ may partly contribute to JMJD2A-medicated promotion of liver cancer cell growth. This assertion is identified by several observations: (1) JMJD2A inhibited P21(WAF1/Cip1) and enhanced Pim1 in liver cancer cell cycle progress through JMJD2AΔ dependent on miR372. (2) JMJD2A promoted cell growth, colony formation ability and cell growth *in vivo*. However, both JMJD2A plus P21WAF1/Cip1 and JMJD2A plus Pim1RNAi abrogated the JMJD2A action. Pim1 and P21(WAF1/Cip1) determine JMJD2A oncogenic function. These findings are noteworthy, given that JMJD2AΔ is a and functions as a key oncogene mediatng various biological processes including cell proliferation, differentiation.

Studies show that CTCF has been found as a regulation factor of gene splicing [35, 36]. In particular, CTCF, the Argonaute protein AGO1, and the heterochromatin protein 1 (HP1) have been indicated in the process of gene splicing [37]. Moreover, HP1 is associated with gene's alternative splicing [38].

In the present study, we clearly indicated that 1)JMJD2A inhibited P21(WAF1/Cip1) in cell cycle progress through JMJD2AΔ dependent on miR372.2)JMJD2A enhanced Pim1 by inhibiting P21(WAF1/Cip1) through JMJD2AΔ dependent on miR372. 3) JMJD2A promotes cell cycle progression via Pim1-pRB-CDK2-CycinE-C-myc pathway, 4)Pim1 and P21(WAF1/Cip1) determine JMJD2A oncogenic function. Evidently, our findings suggest that both Pim1 and P21WAF1/Cip1 determine the JMJD2A function during hepatocarcinogenesis. It is well known P21 (CiP1/WAF1) acts a suppressor in the early stage of cancer invasiveness [39]. Inhibition of p21(WAF1/CIP1) promotes carcinogenesis [40]. Furthermore, lower p21WAF1/cip1 is associated with cancer recurrence and poor prognosis [41, 42]. Pim-1 is promotes cell cycle progression and oncogene transcriptional activation [43, 44]. Moreover, The oncogenic Pim-1 kinase has been implicated as a cofactor for c-MYC and is able to phosphorylate many targets in carcinogenesis [45, 46, 47]. Moreover, Pim-1 is related to various cancers, e.g. prostate carcinomas [48].

Furthermore, our present results show that JMJD2A oncogenic action is associated with Cyclin-dependent kinase 2 (CDK2) and RB activity. It is well known that CDK2 regulates and controls cell cycle progression through several signaling pathway, e.g. EGF—ELK4/c-Fos pathway [49]. Of significance, specific cyclinE/ CDK2 inhibitors can block tumor progression [50]. Futhermore, defects in pRb tumor suppressor may lead to tumorigenesis [51].

### In conclusions

The present study indicates a novel evidence for JMJD2A in tumorigenesis by upregulating miR372 in liver cancer cells, which may have potential therapeutic significance. Alteration of the expression of JMJD2A may also mediate changes to affect gene expression and contribute to hepatocarcinogenesis. JMJD2A knockdown in combination with blocking miR372 might represent a promising treatment strategy targeting tumors with over-activated JMJD2A.
